# The effects and mechanism of urease inhibitor and its combination with nitrification inhibitor on nitrous oxide emission across four soil types

**DOI:** 10.3389/fpls.2025.1663261

**Published:** 2025-09-18

**Authors:** Churong Liu, Benjie Li, Qihua Wu, Diwen Chen, Wenling Zhou, Junhua Ao

**Affiliations:** ^1^ Guangdong Academy of Sciences, Institute of Nanfan and Seed Industry, Guangzhou, China; ^2^ Zhanjiang Research Center, Institute of Nanfan and Seed Industry, Guangdong Academy of Sciences, Zhanjiang, Guangdong, China

**Keywords:** N_2_O emission, urease inhibitor, double inhibitor, nitrogen cycling, metagenomics

## Abstract

Nitrogen (N) fertilization is essential for ensuring crop productivity, while excessive N application significantly increases greenhouse gases (GHGs) emissions, particularly nitrous oxide (N_2_O). Urease inhibitors (UI) and combined urease and nitrification inhibitors (UN) have demonstrated potential in mitigating GHGs emission, though their efficiency with great variation across different soils types. In this study, controlled incubation experiments were conducted using four types of agricultural soils to evaluate the mitigation potential of UI and UN application and to investigate their underlying mechanisms. N fertilization significantly increased N_2_O emissions by 5.1~99.9-fold and elevated CO_2_ emissions by 13.6~65.4% across all soil types. The UI treatment decreased the peak of NO_2_^–^ concentrations in two alkaline soils, while the UN treatment decreased both NO_2_^–^ and NO_3_^–^ concentrations in all four soils. In terms of GHG mitigation, UI treatment reduced N_2_O emissions by 16.5~57.4% in alkaline soils and reduced CO_2_ emissions by 6.5~49.3% across four soil types. The UN treatment demonstrated superior efficacy, reducing N_2_O emissions by 52.5~92.4% and CO_2_ emissions by 4.2~87.2% across all soils. Metagenomic sequencing revealed that both UI and UN significantly inhibited the relative abundances of key functional genes associated with nitrification (*hao* and *nxrAB*), dissimilatory nitrate reduction (*narGHI/napAB*), nitrite reduction (*nirS/nirK*), and nitric oxide reduction (*norBC*). Random forest identified key factors influencing the N_2_O mitigation efficiency of UI and UN. These included soil properties such as soil pH, total nitrogen, organic matter, available potassium, water-filled pore space, texture. Additionally, partial functional genes related to nitrification, denitrification, carbon and methane metabolism, sulfur and phosphorus cycling were also identified as key contributors. Overall, these findings provide valuable insights for the region-specific application of UI and UN to effectively mitigate GHGs emissions. The identification of key soil abiotic and biotic factors offers a theoretical foundation for optimizing inhibitors application and enhancing their mitigation efficiency.

## 1 Introduction

China, possessing only 7.8% of the world’s arable land, accounts for approximately 22.7% of global N fertilizer consumption (FAO, 2024), reflecting a serious problem of excess N fertilization in its agricultural systems. Previous studies have estimated that nearly 50% of the N fertilizer is lost from agricultural soils through various pathways, including ammonia (NH_3_) volatilization, nitrate (NO_3_^–^) leaching and runoff, and nitrous oxide (N_2_O) emissions. These reactive N losses not only result in substantial agronomic and economic damages but also cause considerable environmental burdens (Galloway et al., 2008; Coskun et al., 2017). Among these N loss pathways, N_2_O is of particular contaminant due to its dual role as a potent greenhouse gas and an ozone-depleting substance. With an atmospheric lifetime of approximately 116 years and a global warming potential 273 times greater than that of carbon dioxide (CO_2_), N_2_O is major contributor to both stratospheric ozone depletion and climate warming (Tian et al., 2020; IPCC, 2021). Globally, an estimated approximately 60% of anthropogenic N_2_O emissions originate from agricultural soils, where microbial processes such as nitrification, nitrifier denitrification, denitrification, and dissimilatory nitrate reduction to ammonium (DNRA) drive N_2_O production (Reay et al., 2012). Among these, nitrification and denitrification are widely recognized as the dominant biological pathways, accounting for around 70% of N_2_O emissions from soils (Butterbach-Bahl et al., 2013; Wrage-Mönnig et al., 2018). Excessive N fertilization not only intensifies N_2_O emissions but may also stimulate the release of other greenhouse gases (GHGs), such as CO_2_ and CH_4_. Therefore, the identification of effective N_2_O mitigation strategies, along with a comprehensive understanding of their efficacy across diverse soil and environmental conditions, is critical for improving N use efficiency and mitigating the agricultural GHGs emissions from agricultural systems.

Nitrification inhibitors (NIs) reduce reactive N losses primarily by suppressing the nitrification process. Currently, the most widely commercialized NIs include 3,4-dimethylpyrazole phosphate (DMPP), 2-chloro-6-(trichloromethyl)pyridine (CP), and dicyandiamide (DCD). DMPP is the most extensively used due to its lower application rates, limited mobility in soil, and higher environmental safety compared to CP and DCD (Gao et al., 2021; Liu et al., 2021). DMPP is reported to inhibit ammonia oxidation process by competitively binding to the copper-binding site on the AmoB subunit, thereby influencing soil N transformations and related microbial communities (Zerulla et al., 2001). Numerous studies have shown that the application of NIs in agricultural soils can increase N recovery efficiency by 4~93% and crop yields by 6~13%, while reducing N_2_O emissions by 8~94%. However, the mitigation efficiency of NIs varies considerably across agroecosystems due to differences in soil type, climate, and management practices (Qiao et al., 2015; Lam et al., 2017; Liu et al., 2022a). Urease inhibitors (UIs), such as N-(n-butyl) phosphoric triamide (NBPT), N-(propyl) thiophosphoric triamide (NPPT), and hydroquinone (HQ), function by delaying the hydrolysis of urea. This delay helps moderate the transient pH spike caused by rapid urea delaying, thereby reducing the NH_3_ volatilization and other reactive N losses (Cantarella et al., 2018). UIs application has been shown to enhance N use efficiency by 14~29% and crop yields by 3~10%, while reducing N_2_O emissions by 2~77% (Li et al., 2018; Liu et al., 2022b). Given the multiple pathways through which reactive N loss in field conditions, the combined application of NIs and UIs (double inhibitors, UNs) is gaining increasing attention. However, compared with the relatively extensive studies on NIs, investigations into the effects of UIs and UNs on N_2_O emissions remain limited, possibly due to differences in their modes of action and design rationale.

Various inhibitors offer a simple and operationally feasible strategy for N_2_O mitigation, especially when compared to conventional strategies such as deep fertilizer placement, precision fertilization, or integrated agronomic management, which often demand specialized equipment and technical expertise. These advantages render inhibitor-based strategies particularly suitable for large-scale promotion in China’s smallholder-dominated agricultural systems (Xia et al., 2017). Previous studies have demonstrated that NIs primarily function by suppressing the activity and abundance of ammonia-oxidizing bacteria (AOB), and under certain conditions, NIs also influence ammonia-oxidizing archaea (AOA) as well as the abundance of other N-cycling microorganisms and functional genes (Fan et al., 2019; Lu et al., 2019; Liu et al., 2021). However, DMPP has generally shown negligible effects on the overall structure and function of the soil microorganism (Liu et al., 2023a). In alkaline soils, NBPT has been reported to stimulate the relative abundance of nitrification-related genes while simultaneously reducing the abundance of genes involved in denitrification processes (Liu et al., 2022a). The variation in N_2_O mitigation efficiency of inhibitors is primarily attributed to the complex interactions between climatic conditions (e.g., rainfall, temperature), soil physicochemical properties (e.g., pH, organic matter, total N, available phosphorus, and potassium) and microbial communities and functions, which influence both biotic and abiotic N transformation processes (Lam et al., 2017; Liu et al., 2023b, 2023c). These differences also reflect the ecological divergence among various types of N-cycling microorganisms (Yin et al., 2022). Previous studies indicated that the NIs efficiency in N_2_O mitigation is governed by the interactions between soil physicochemical properties, microbial community composition, and functional gene expression (Shi et al., 2016; Liu et al., 2023b). However, the relative contributions and mechanisms of abiotic and biotic factors in regulating the N_2_O mitigation efficiency of UIs and UNs remain poorly understood. This knowledge gap constrains the broader deployment of inhibitors application and limits their mitigation potential in large-scale agricultural systems.

To investigate the underlying mechanisms of variable efficiency of UI and UN on N_2_O mitigation. This study conducted controlled microcosm experiments using four distinct agricultural soil types (i.e., dark brown, fluvo-aquic and red soil), which were selected based on pronounced differences in physicochemical properties, particularly soil pH, organic matter content, and particle size distribution, as these factors are considered potential drivers of variation in inhibitor efficiency. Through dynamic observation of inorganic N transformations and N_2_O emissions, and integrating 16S-rRNA amplicon sequencing, metagenomic sequencing, and random forest modeling, we aimed to address the following objectives: (i) to evaluate the effects of UI and UN applications on N transformations and N_2_O emissions across different soil types; (ii) to identify the key abiotic and biotic factors, as well as the underlying mechanisms, that regulate the N_2_O mitigation efficiency of UI and UN.

## 2 Materials and methods

### 2.1 Study site of soil sample and experimental design

Soil samples for the incubation experiments were collected from four representative cropland across China. The first site, Gongzhuling Station (Jilin Province, JL, 43°40’N, 124°67’E), is located in a humid to semi-humid continental monsoon region. The surface soil is classified as dark brown soil with a clay loam texture. Its physicochemical characteristics include a pH of 6.4, soil organic matter (SOM) content of 65.9 g kg^–1^, total nitrogen (TN) of 1.8 g kg^–1^, available phosphorus (AP) of 40.8 mg kg^–1^, and available potassium (AK) of 145.8 mg kg^–1^. The second site, Wuqiao Station (Hebei Province, HB, 37°40’N, 116°38’E), lies in a semi-arid continental monsoon climate zone. The soil is classified as fluvo-aquic soil with a sandy clay loam texture, exhibiting a pH of 7.7, and the SOM, TN, AP, and AK contents are 24.9 g kg^–1^, 1.0 g kg^–1^, 18.8 mg kg^–1^, and 182.1 mg kg^–1^, respectively. The third site, Yanjin Station (Henan Province, HN, 35°20’N, 114°12’E), is also situated in a semi-arid continental monsoon climate. The topsoil is categorized as fluvo-aquic with a sandy loam texture. Soil pH value is 7.8, and SOM, TN, AP, and AK are 18.4 g kg^–1^, 0.8 g kg^–1^, 15.2 mg kg^–1^, and 75.2 mg kg^–1^, respectively. The fourth sampling location is Tengqiao Station (Zhejiang Province, ZJ, 28°10’N, 120°51’E), which experiences a typical oceanic monsoon climate. The soil is classified as red soil with a clay loam texture, showing a pH, SOM, TN, AP, and AK are 4.4, 38.1 g kg^–1^, 1.2 g kg^–1^, 49.2 mg kg^–1^ and 98.2 mg kg^–1^, respectively. At each location, fifteen surface soil cores (0–20 cm depth) were collected using a soil auger and thoroughly homogenized to form one composite sample per location. All composite samples were passed through a 2-mm sieve and stored at 4 °C until further use in incubation experiments.

Four fertilizer treatments were applied to each of the four soil types (JL, HB, HN, and ZJ), resulting in a total of 16 treatment combinations: (1) no N fertilizer (Control, Con); (2) urea alone (U); (3) urea combined with NBPT (UI); (4) urea supplemented with both NBPT and DMPP (UN). GHGs samples were monitored using a closed static incubation system, which consisting of 650 mL glass bottles sealed with rubber stoppers equipped with link valves. Soil samples were collected from a soil incubation system, which consists of 500 ml glass bottle and gas-permeable membranes. For each system, 100 and 50 g dry weight of soil was added to gas and soil incubation system. Prior to the incubation experiment, all soil samples were pre-incubated for 7 days at 25 °C under 50% water-filled pore space (WFPS) to stabilize microbial activity and minimize disturbance effects. All incubations were conducted for 28 days at 25 °C and maintained at 60% WFPS. N fertilizer was applied at a rate of 100 mg N kg^-1^ dry soil, while the application rates of NBPT and DMPP were 0.08% and 1.0% of the applied urea-N, respectively.

GHGs samples were collected on days 1, 2, 3, 5, 7, 9, 14, 21, and 28 of the incubation periods. For each sampling time, Headspace air of 30 mL was extracted from gas incubation system and injected into an evacuated vial. Following sampling, bottles were uncapped for at least 15 minutes to allow sufficient air exchange before resealing. Soil samples for inorganic N analysis (10 g fresh weight) were destructively sampled on days 1, 3, 7, 14, and 28, and immediately stored at –20 °C until analysis. For microbial analysis, soils were destructively sampled on day 14 and stored at –80 °C prior to DNA extraction. All treatments were conducted in triplicate. In total, 336 incubation bottles were prepared: 48 for gas sampling (4 soil types × 4 treatments × 3 replicates), 240 for inorganic N analysis (4 soil types × 4 treatments × 3 replicates × 5 time points), and 48 for microbial DNA extraction (4 soil types × 4 treatments × 3 replicates × 1 time point).

### 2.2 Greenhouse gases and soil analysis

GHGs (N_2_O, CO_2_, and CH_4_) samples were analyzed by gas chromatography (Agilent 7890A GC; Agilent, USA). The GHGs mitigation efficiency of urease inhibitor (UI) and double inhibitor (UN) was computed equation: Mitigation efficiency = (U treatment – UI/UN treatment)/(U treatment – Con treatment), wherein Con, U, UI, UN treatments were GHGs accumulation of corresponding treatment. Soil texture was characterized by the sedimentation methods based on particle size distribution. Soil pH was measured in a 1:2.5 (w/v) soil-to-deionized water suspension using a calibrated pH meter (FE28; Mettler Toledo, USA). Total nitrogen (TN) and soil organic matter (SOM) were determined via the Kjeldahl digestion method (Kjeltec 8400; FOSS, Denmark) and potassium dichromate oxidation, respectively. Soil inorganic N (NH_4_^+^-N, NO_2_^−^-N, and NO_3_^−^-N) was extracted using 2 M KCl solution at a soil-to-solution ratio of 1:5 (w/v), with shaking for 1 hour. The achieved extracts were analyzed using a continuous flow injection analyzer (Auto Analyzer 3; SEAL Analytical, USA). The computed equation of WFPS was according to Liu et al. (2023b). Available phosphorus (AP) and available potassium (AK) were quantified using the sodium bicarbonate extraction method and the ammonium acetate extraction method, respectively, as described by Lu (1999).

### 2.3 DNA extraction and high-throughput sequencing

Genomic DNA was extracted from 0.5 g of fresh soil using the FastDNA^®^ Spin Kit for Soil (MP Biomedicals, CA, USA) following the manufacturer’s protocol. The concentration and purity of the extracted DNA were assessed using a Nanodrop 2000 spectrophotometer (Thermo Fisher Scientific, MA, USA), and DNA integrity was verified by electrophoresis on a 1.5% agarose gel. High-quality DNA samples were stored at –80 °C until subsequent microbial sequencing, which was performed by Majorbio Bio-Pharm Technology Co., Ltd. (Shanghai, China).

DNA sample was divided into two subsamples, one subsample was used for high-throughput sequencing of universal bacterial 16S rRNA, and the other part was used for sequencing of the metagenome. The V3–V4 regions (Liu et al., 2016) of the bacterial 16S rRNA gene were amplified using primers 338F (5’-ACTCCTACGGGAGGC AGCAG-3’) and 806R (5′-GGACTACNNGGGTATCTAAT-3′). PCR amplification was performed using a high-fidelity DNA polymerase under standard thermal cycling conditions. Amplicons were purified, quantified, and pooled in equimolar concentrations. Paired-end sequencing was conducted on the Illumina MiSeq PE250 platform (Illumina, San Diego, CA, USA) by Majorbio Bio-Pharm Technology Co., Ltd. (Shanghai, China). Raw sequence data were demultiplexed using the fastp (version 0.20.0) and merged by FLASH (version 1.2.7) (Magoč and Salzberg, 2011; Chen et al., 2018). After demultiplexing, reads were quality-filtered, trimmed, and denoised using the UPARSE 7.1 to generate operational taxonomic units (OTUs) with 97% sequence similarity level (Edgar, 2013). Taxonomic classification was performed against the SILVA 138 reference database using of national center for biotechnology information (NCBI) with confidence threshold of 0.7.

For metagenomic analysis, high-quality genomic DNA was randomly fragmented to an average insert size of 400 bp using a Covaris M220 (Gene Company Limited, China). Libraries were constructed using the NEBNext^®^ Ultra™ DNA Library Prep Kit (New England Biolabs, USA) and sequenced on an Illumina NovaSeq platform (Illumina, San Diego, CA, USA). Raw reads were subjected to quality control using fastp (version 0.20.0), including removal of adapter sequences, low-quality reads, and reads with ambiguous bases. Clean reads were assembled *de novo* into contigs using MEGAHIT (version 1.2.9, Li et al., 2015). Open reading frames (ORFs) were predicted using MetaGene (Noguchi et al., 2006), and non-redundant gene catalogs were constructed using CD-HIT (Fu et al., 2012) with 90% sequence identity and 90% coverage. Functional annotation of genes involved in carbon (C), nitrogen (N), phosphorus (P), and sulfur (S) cycling was conducted by aligning sequences against the Kyoto Encyclopedia of Genes and Genomes (KEGG) database using DIAMOND (Buchfink et al., 2015) with an e-value cutoff of 1e^-5^.

### 2.4 Bioinformatics analysis

Alpha diversity indices, including Shannon and Chao1, were computed to assess microbial taxonomic and functional diversity across different treatments. Beta diversity was evaluated using principal coordinate analysis (PCoA) based on Bray-curtis dissimilarity matrices, implemented through the “vegan” package in R version 4.5.0 (Robert and Ross, Auckland, NZ). The permutational multivariate analysis of variance (PERMANOVA) were conducted to assess significant differences in microbial and functional composition among treatments. To investigate the influence of abiotic and biotic factors on the N_2_O mitigation efficiency of inhibitors, random forest analysis was performed using the “rfPermute” package. The model’s explanatory power and statistical significance were assessed by calculating the coefficient of determination (R²) and p-values using the “A3” package in R (Liu et al., 2023c).

### 2.5 Statistical analysis

All statistical analyses were conducted using SPSS software (version 18.0; IBM. Armonk, NY, USA). Differences in soil inorganic N contents, GHGs accumulation, and alpha diversity indices among treatments were assessed using one-way analysis of variance (ANOVA). The least significant differences (LSD) test at *p* < 0.05 was considered to indicate significant differences. Line, and histogram bar graphs were created using SigmaPlot 12.5 (Systat, San Jose, USA) to visualize treatment effects and data trends.

## 3 Results

### 3.1 Dynamics of inorganic nitrogen

**Figure 1:**
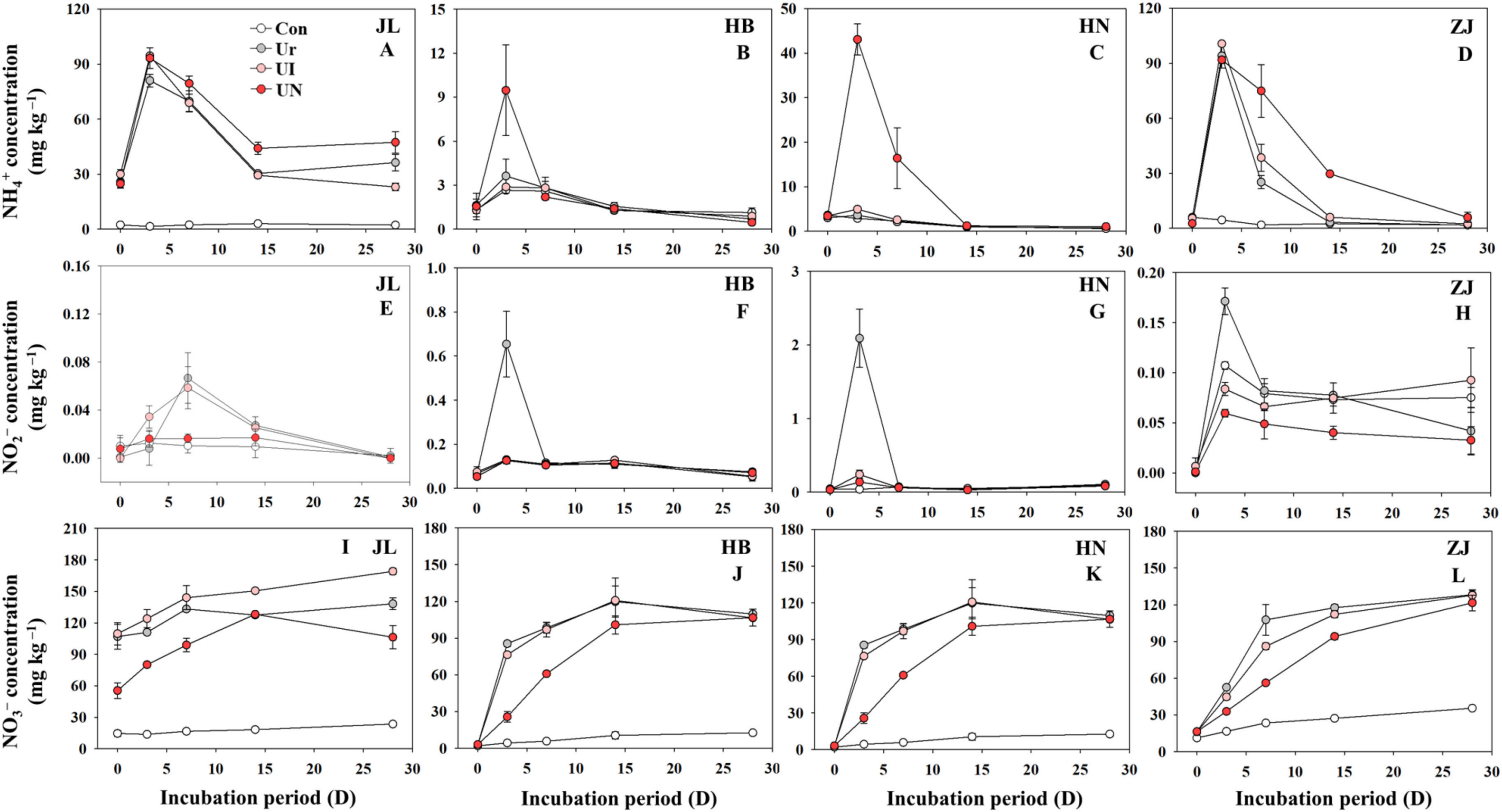
Dynamics of soil NH_4_^+^ **(A–D)**, NO_2_^–^ **(E–H)**, and NO_3_^–^ **(I–L)** during the incubation period.

The temporal dynamics of the three forms of inorganic N exhibited significant variation. Soil type playing a pivotal role in shaping both the concentrations of inorganic N and the inhibitors effectiveness (**Figure 1**). The peak of NH_4_+–N concentration occurred on day 3 after fertilization, which showed markedly higher peaks observed in acidic soils (JL and ZJ soils) than alkaline soils (HN and HB soils). Compared to the U treatment, The UI treatment demonstrated less impact on NH_4_+–N concentration, while the UN treatment obviously increased NH_4_+–N concentrations in alkaline soils (**Figures 1A–D**). The peak of NO_2_−–N concentration between days 3~7 after fertilization, and were substantially higher in alkaline soils than in acidic soils. Relative to the U treatment, both UI and UN treatments markedly decreased NO_2_−–N concentration in alkaline soils, while showed negligible effects in acidic soils (**Figures 1E–H**). NO_3_−–N, the most stable form of inorganic N in soil, which exhibited a steady increase throughout the incubation period, generally reaching maximum value around day 14 after fertilization (**Figures 1I–L**). The UI treatment showed minimal influence on NO_3_−–N concentration, whereas the UN treatment effectively reduced NO_3_−–N concentration compared to the U treatment.

### 3.2 GHGs emission

**Figure 2:**
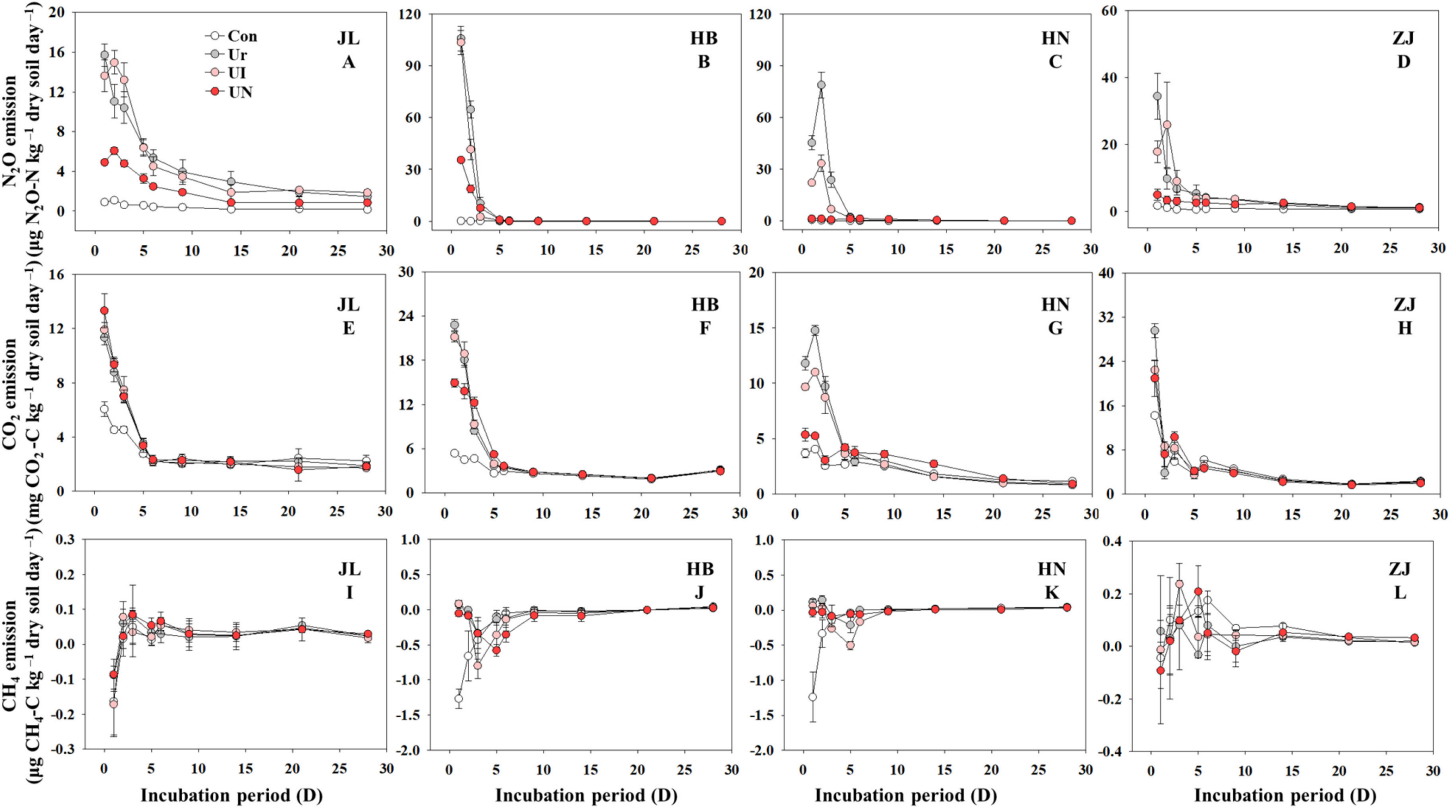
Dynamics of N_2_O **(A–D)**, CO_2_ (**(E–H)**), and CH_4_ (**(I–L)**) emission during the incubation period. Con indicated no N fertilizer; Ur indicated urea alone application; UI indicated urea combined with NBPT application; UN indicated urea supplemented with both NBPT and DMPP application.

**Figure 3:**
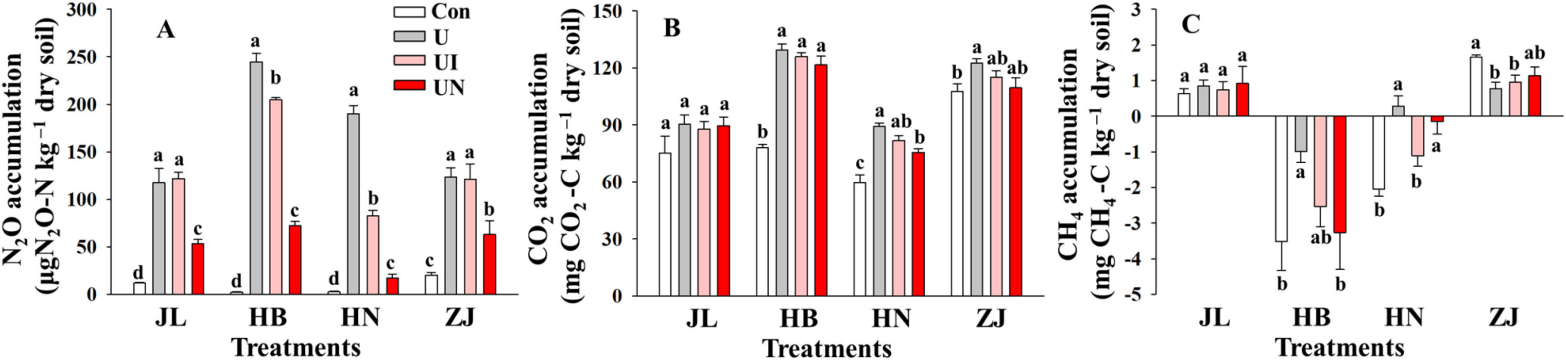
Cumulative emissions of N_2_O **(A)**, CO_2_ **(B)**, and CH_4_ **(C)** during the incubation period. Con indicated no N fertilizer; Ur indicated urea alone application; UI indicated urea combined with NBPT application; UN indicated urea supplemented with both NBPT and DMPP application. Different lowercase letters indicate significant differences between the treatments (p < 0.05).

GHGs emissions intensively occurred within the first 7 days after N fertilization, after which emissions remained low and relatively stable throughout the remainder of the incubation period. Both CO_2_ and N_2_O exhibited peak emissions within day 1~3 days post-fertilization, while CH_4_ emissions showed erratic and inconsistent trends (**Figure 2**). N fertilization exerted markedly different effects on the three GHGs (**Figure 3**). The U treatment significantly increased cumulative N_2_O emissions by 5.1~99.9-folds and elevated CO_2_ emissions by 13.6~65.4%. In contrast, the impact of N fertilization on CH_4_ emissions varied by soil type, showing increases, decreases, or no significant changes. The UN treatment was the most effective in suppressing N_2_O emissions, which decreasing by 52.5~92.4% across the four soil types. The UI treatment significantly decreased 16.5~57.4% only in alkaline soils but had no discernible effect in acidic soils. With respect to CO_2_ emissions, the U treatment significantly enhanced CO_2_ emissions by 13.6~65.4%. In comparison, the UI and UN reduced CO_2_ emissions by 6.5~49.3% and 4.2~87.2%, respectively. CH_4_ emissions responded inconsistently to inhibitor treatments. Compared to the U treatment, the UI treatment reduced CH_4_ emissions in JL, HB, and HN soils, and the UN treatment promoted CH_4_ uptake in HB and HN soils. The use of inhibitors tended to increase CH_4_ emissions except the above situations.

### 3.3 Diversity of microbial community and function

**Figure 4:**
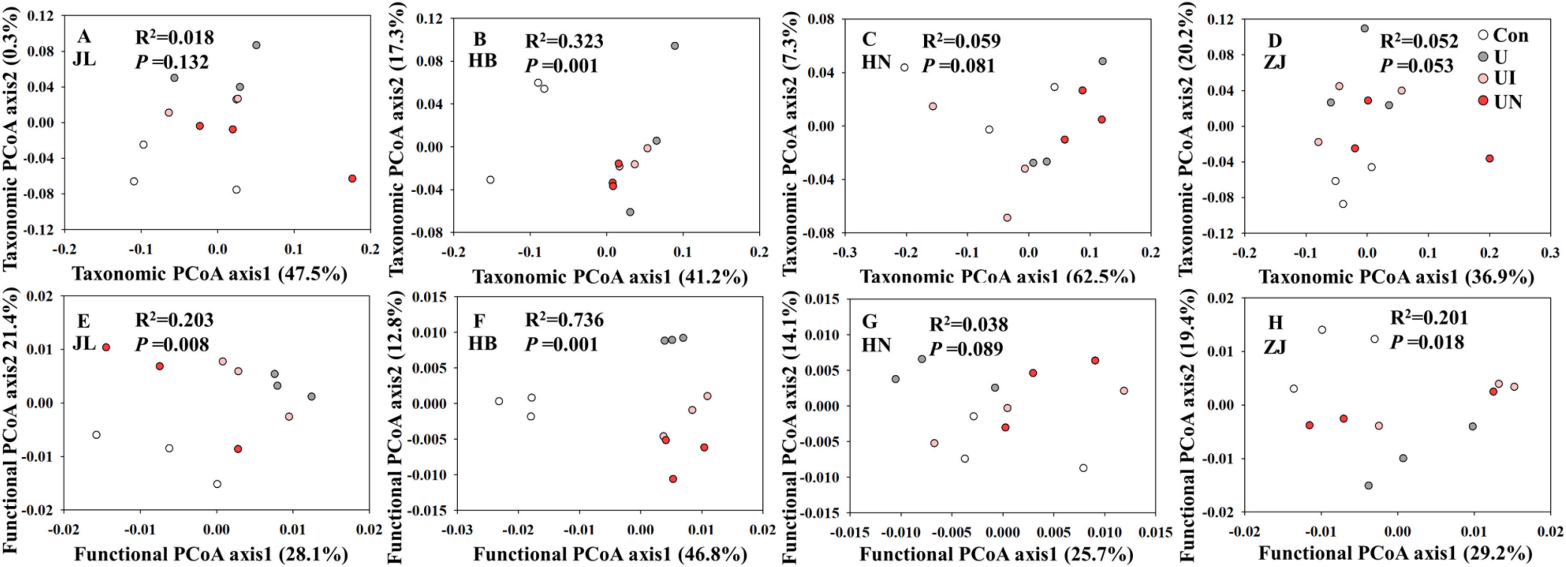
Principal coordinates analysis (PCoA) analysis of microbial community (genus level, **A–D**) and function (KO level, **E–H**) for all treatments. Con indicated no N fertilizer; Ur indicated urea alone application; UI indicated urea combined with NBPT application; UN indicated urea supplemented with both NBPT and DMPP application.

In this study, the application of inhibitors (UI and UN) exerted minimal effects on both α and β diversity at the genus level (**Supplementary Table S1**, **Figures 4A–D**). In HB soil, the U treatment significantly reduced the Chao1 richness index of microbial genera, whereas the UI and UN treatments effectively mitigated this decline. In HN soil, the U treatment decreased the Shannon diversity index, while the UI treatment slightly increasing it and the UN treatment further reducing it, and these changes were not statistically significant (**Supplementary Table S1**). Both N and inhibitors application significantly altered the microbial community composition in HB soil (**Figure 4B**; PERMANOVA, *p* < 0.05). Across all four soil types, N fertilization increased the Chao1 index of microbial function, and this effect was further enhanced by UI and UN application, although the differences were not statistically significant (**Supplementary Table S1**). In contrast, no significant changes were observed in the Shannon index of microbial functions in response to N or inhibitor treatments. Both N and inhibitors application significantly modified the functional structure of microorganism in JL, HB, and ZJ soils (**Figures 4E, F, H**; PERMANOVA, *p* < 0.05), with the exception of HN soils (**Figure 4G**). Overall, inhibitor treatments had relatively minor effects on the diversity of microbial communities and their functions. Therefore, it is more important to pay attention to the changes of specific functional genes.

### 3.4 Change of nitrogen and nutrient cycling genes

**Figure 5:**
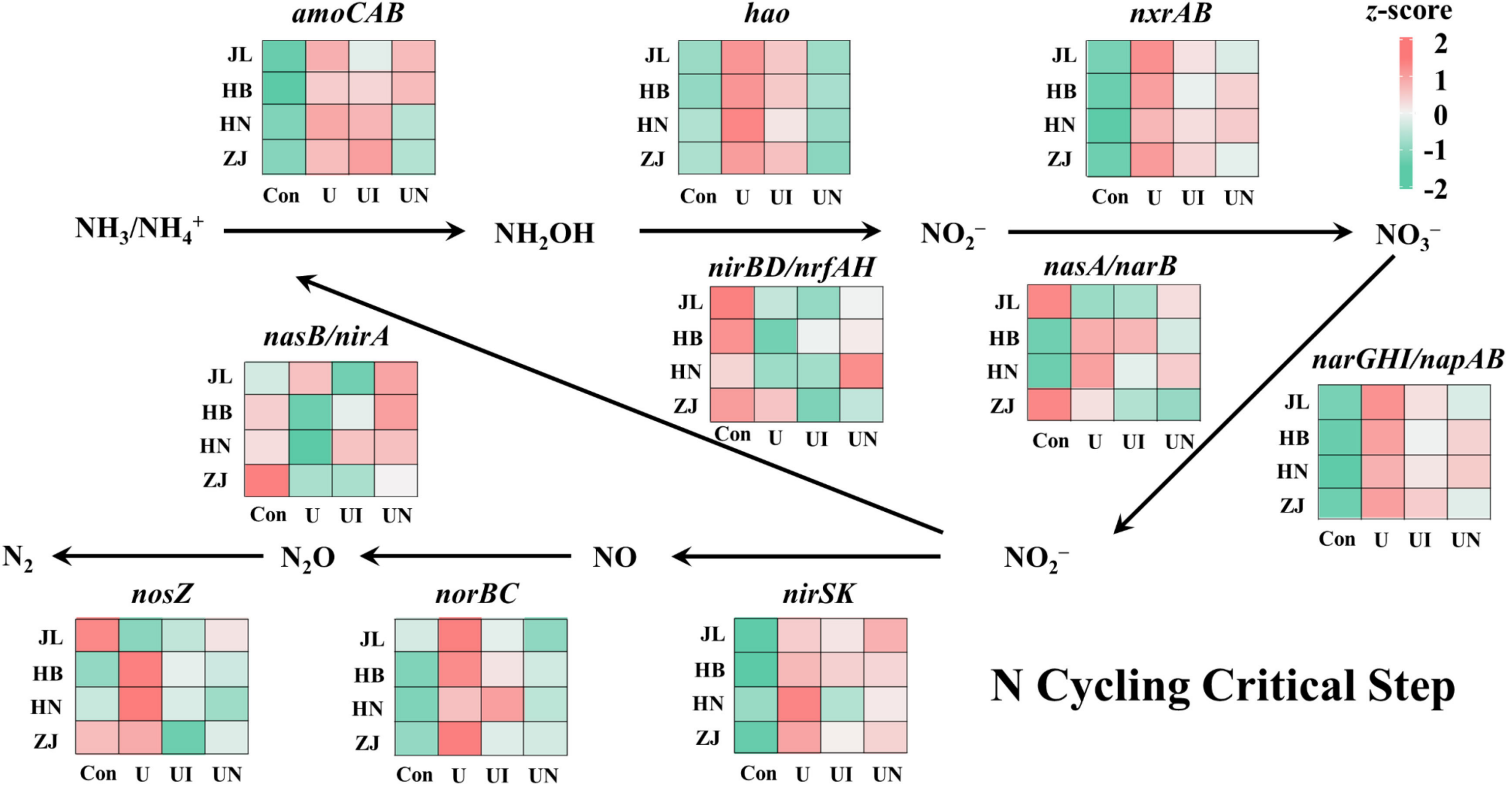
The change of key functional genes of nitrogen cycling for all treatments. Con indicated no N fertilizer; Ur indicated urea alone application; UI indicated urea combined with NBPT application; UN indicated urea supplemented with both NBPT and DMPP application.The effects of different treatments on C fixation, C metabolism, CH_4_ metabolism, P cycling, and S cycling are presented in **Supplementary Figure S1**. Notable variations in the relative abundance of key functional genes involved in C, P, and S cycling were observed across the different soil types. N fertilization significantly altered the relative abundance of these functional genes, while their responses to UI or UN treatments were more variable.

N fertilization significantly influenced the relative abundance of functional genes associated with key nutrient cycles (i.e. carbon, nitrogen, phosphorus, and sulfur cycles), with the most pronounced effects observed for nitrogen cycling genes (**Figure 5**). The U treatment significantly enhanced the abundance of nitrification-related genes, with *amoCAB*, *hao*, and *nxrAB* genes increased by 20.1~73.3%, 111.1~359.6%, and 11.9~17.3%, respectively. In JL, HB, and HN soils, the UI treatment reduced relative abundance of *amoCAB* gene by 6.1~44.8% relative to U treatment, whereas in ZJ soil, UI treatment resulted in a 21.3% increase in relative abundance of *amoCAB* gene. The UN treatment decreased relative abundance of *amoCAB* gene by 7.1~73.4% in JL, HN, and ZJ soils, while caused a 10.5% increase in HB soil. Across four soil types, both UI and UN treatments consistently decreased relative abundance of *hao* and *nxrAB* genes compared to U treatment. Specifically, the UI treatment decreased *hao* and *nxrAB* by 25.4~59.1% and 21.1~46.5%, respectively, while the UN treatment led to reductions of 89.9~120.0% for *hao* and 11.2~50.2% for *nxrAB*. Overall, N fertilization promoted the relative abundance of genes involved in nitrification, whereas inhibitors application significantly reduced these gene, with *hao* showing the most consistent response across all soil types. In contrast, the denitrification pathway exhibited more diverse responses due to its regulation by a broader range of functional genes. N application increased the relative abundance of genes involved in dissimilatory nitrate reduction (*narGHI/napAB*), nitrite reduction (*nirS/nirK*), and nitric oxide reduction (*norBC*) across four soils. Both the UI and UN treatments effectively attenuated the N-induced increases in relative abundance of these genes. However, no consistent pattern was observed for genes involved in dissimilatory nitrate reduction to ammonium (*nasB/nirA* and *nirA/nasB*) and nitrous oxide reduction (*nosZ*). This suggests that microorganisms which carrying the relevant functional genes might be remarkable differences in disparate soil.

### 3.5 Potential impact factors for inhibitors efficiency of N_2_O mitigation

**Figure 6:**
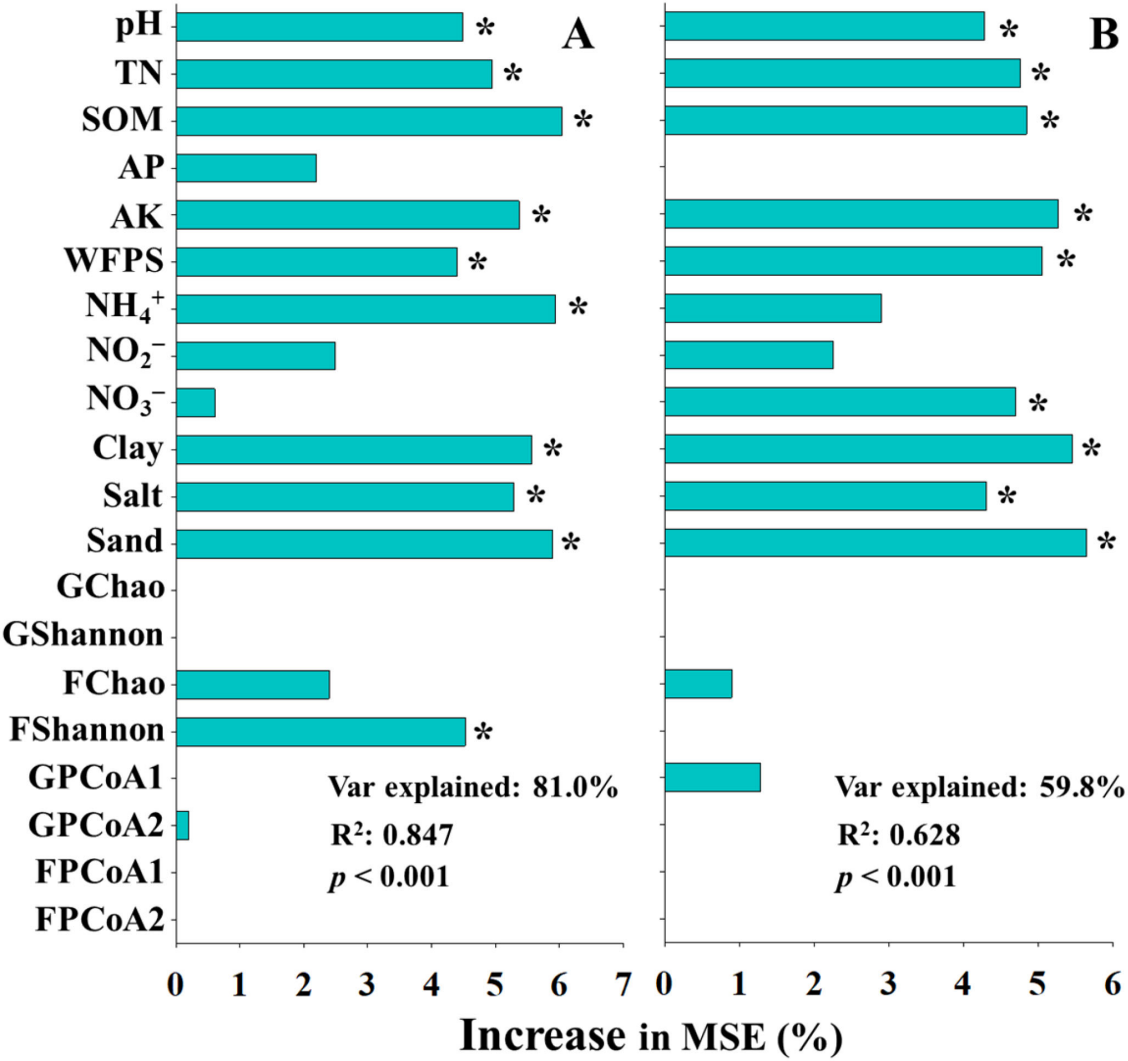
The predictions of main soil and microbial factors to N_2_O mitigation efficiency of urease **(A)** and double inhibitor **(B)** base on random forest analysis. SOM, soil organic matter; TN, total nitrogen; AP, available phosphorus; AK, available potassium; WFPS, water-filled pore space; GChao, GShannon. GPCoA1, and GPCoA2 indicated taxonomic α and β diversity, FChao, FShannon. FPCoA1, and FPCoA2 indicated functional α and β diversity. *indicated significant differences at *p* < 0.05.

Given the superior performance of inhibitors in mitigating N_2_O emissions, random forest (RF) modeling was employed to explore the contribution patterns of environmental, soil physicochemical, and microbial factors to the N_2_O mitigation efficiency of inhibitors (**Figure 6**). For the UI treatment, soil pH, TN, SOM, AK, WFPS, NH_4_^+^ concentration, clay content, salt content, sand content, and functional Shannon index were identified as significant predictors of N_2_O mitigation efficiency (**Figure 6A**). In contrast, for the UN treatment, soil pH, TN, SOM, AK, WFPS, NO_3_^–^ concentration, clay content, salt content, and sand content were recognized as significant contributors (**Figure 6B**). The model for UI efficiency explained a higher explanatory weight and exhibited better model fit (Var explained: 81.0%, R^2^ = 0.847) than the model for UN efficiency (Var explained: 59.8%, R^2^ = 0.628), suggesting that traditional physicochemical parameters might exert a stronger influence on inhibitors efficiency than microbial diversity indices.

**Figure 7:**
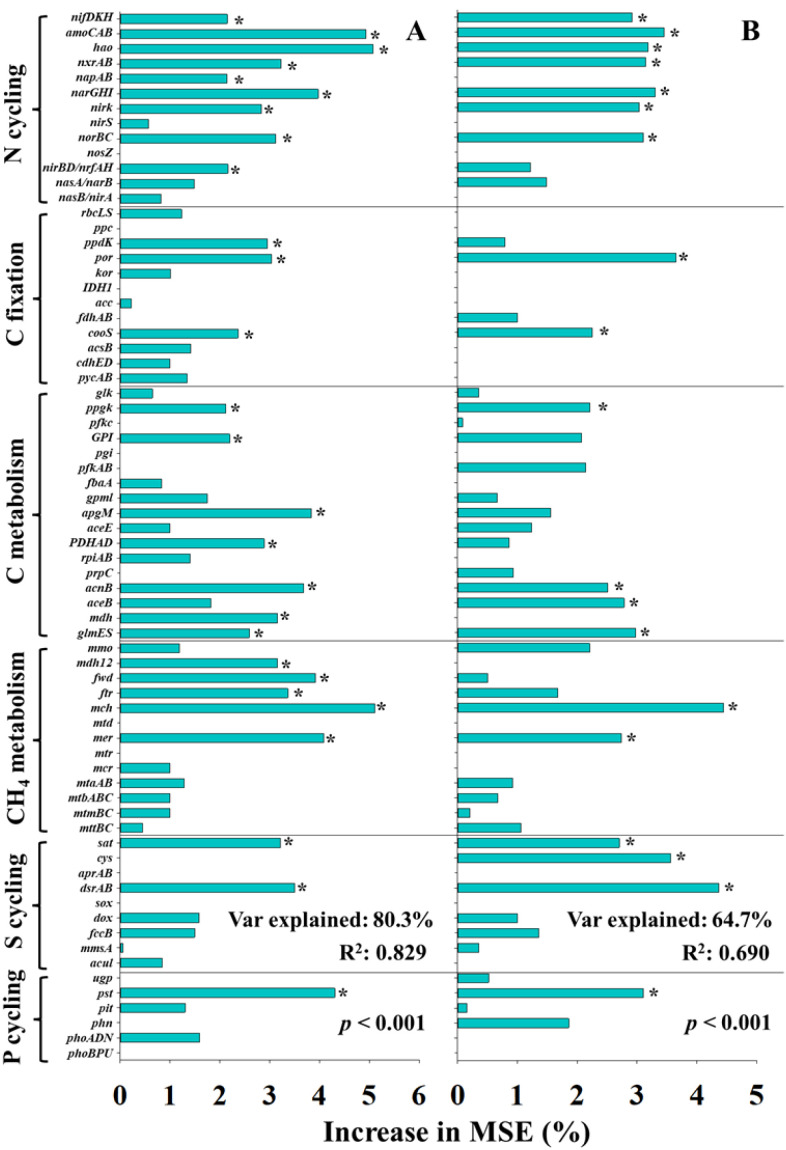
The predictions of main soil and microbial factors to N_2_O mitigation efficiency of urease **(A)** and double inhibitor **(B)** base on random forest analysis. * indicated significant differences at *p* < 0.05.

To further elucidate the specific microbial contributions, nutrient cycling genes were evaluated by RF models (**Figure 7**). For the UI efficiency, key predictors included genes involved in N fixation (*nifDHK*), nitrification (*amoCAB*, *hao*, and *nxrAB*), denitrification (*napAB*, *narGHI*, and *norBC*), dissimilatory nitrate reduction to ammonium (*nirBD/nrfAH*), carbon fixation (*ppdK*, *por*, and *cooS*), C metabolism (*ppgK*, *GPI*, *apgM*, *PDHAD*, *acnB*, *mdh*, and *glmES*), CH_4_ metabolism (*mdh12*, *fwd*, *ftr*, *mch*, and *mer*), S cycling (*sat* and *dsrAB*), and P cycling (*pst*) (**Figure 7A**). In comparison, for the UN efficiency, fewer functional genes showed significant importance, including genes related to N fixation (*nifDHK*), nitrification (*amoCAB*, *hao*, and *nxrAB*), denitrification (*narGHI* and *norBC*), carbon fixation (*por* and *cooS*), C metabolism (*ppgK*, *acnB*, *aceB*, and *glmES*), CH_4_ metabolism (*mch* and *mer*), S cycling (*sat*, *cys*, and *dsrAB*), and P cycling (*pst*) (**Figure 7B**). Overall, RF models of nutrient cycling genes exhibited higher explanatory weight and model fit compared to those based on environmental parameters and microbial diversity indexes, underscoring the central role of functional genes in regulating the efficiency of N_2_O mitigation by UI and UN application.

## 4 Discussions

Reducing reactive N losses from agricultural soils is an effective approach to improving crop N use efficiency, yield, and ecological-economic benefits. This study found that the inhibitors (UI and UN) efficiency significantly varies across different soil types, with both biotic and abiotic soil factors playing crucial roles in determining their efficiency. Such variability leads to inconsistent ecological and economic outcomes from inhibitor application, thereby limiting their scalability and benefits in large-scale agricultural systems.

Soil pH is one of the most critical factors influencing the inhibitors efficiency. Inhibitors (i.e., UI and UN) tend to exhibit greater efficiency in alkaline soils, whereas their efficiency is substantially reduced or even negligible under acidic conditions (Shi et al., 2017; Liu et al., 2023b). It is widely recognized that in alkaline soils, nitrification and nitrifier denitrification are the dominant processes driving N_2_O emissions (Wu et al., 2018), while incomplete denitrification plays the primary role in acidic soils (Li et al., 2020; Khan et al., 2022). Consistent with these studies, our study shows that UI application effectively decreased N_2_O emissions in alkaline soils, whereas its effect is diminished in acidic soils. In contrast, UN application significantly decreased N_2_O emissions across all soil types, although the mitigation efficiency was lower under acidic conditions. For the UI treatment, the observed reduction in peak NO_2_− concentrations suggest a potential mechanism of N_2_O mitigation. This effect was particularly evident in HB and HN soils, where UI significantly decreased NO_2_− peaks. These results support the proposed mechanism by which UI inhibits urease activity by binding to its nickel active site, thereby slowing urea hydrolysis and reducing the availability of nitrification substrates and hydroxylamine oxidation rates, ultimately mitigating N_2_O emissions from nitrification (Krol et al., 2020). However, UI application exhibited less effects on the all kinds of inorganic N in JL and ZJ soils, indicating a weak influence on soil N cycling and fails to decrease N_2_O emissions in acidic soils (Hube et al., 2017). Moreover, the invalidation of UI on N_2_O mitigation also due to UI mitigates nitrification primarily through substrate limitation, and nitrification is not the dominant N_2_O-producing pathway in acidic soils. Therefore, the limited nitrification in acidic systems likely constrains the effectiveness of UI-based mitigation strategies.

The UN application exhibits a stronger regulatory effect on inorganic N transformation and N_2_O emissions compared to the application of UI alone. The UN treatment consistently increased NH_4_^+^ concentrations while significantly decreasing NO_2_− and NO_3_− levels across all soil types. This effect is attributed to the combined mechanisms of UI, which limits the availability of substrates for nitrification (Cantarella et al., 2018), and NI which suppresses both ammonia and nitrite oxidation (Wu et al., 2018). Notably, the N_2_O mitigation efficiency of UN in this study slightly exceeded that reported for NI alone in previous studies (Liu et al., 2023b), indicating a synergistic interaction between the two inhibitors. The mechanisms by which the two inhibitors function are complementary rather than conflicting. Such synergy appears to enhance the control of N transformations in soil and contributes more effectively to the suppression of N_2_O emissions (Ni et al., 2018; Luchibia et al., 2020). However, previous study has reported that although the combined application of DMPP and NBPT can significantly enhance crop yield and N use efficiency, it may exhibit antagonistic effects on N_2_O mitigation in field condition (Zhao et al., 2017). This discrepancy highlights the substantial influence of complex interactions between climatic conditions and soil physicochemical properties on the UN efficiency. Therefore, further studies are needed to investigate the effects of UN on soil nutrient cycling and N_2_O emissions under more diverse and field environmental conditions.

SOM is also a key edaphic factor influencing the inhibitors efficiency. Higher SOM levels are typically associated with greater nutrient availability and stronger sorption capacity (Mehnaz et al., 2019). Elevated nutrient content can promote the richness and diversity of soil microorganism and potential function (Wardle et al., 2008; Farrell et al., 2013), thereby increasing the complexity and heterogeneity of microbially mediated biogeochemical processes, including nutrient cycling and greenhouse gas emissions (Chang et al., 2020). As these processes become more intricate, they are less amenable to regulation by single classes of chemical inhibitors. Moreover, the stronger sorption capacity of high SOM soils may lead to the immobilization of small-molecule inhibitors (i.e. DMPP or NBPT) by adsorptive action of organic matrices, thereby reducing their bioavailability and functional efficacy (Tian et al., 2016). Additionally, SOM provides readily available carbon and nitrogen sources that can stimulate microbial populations capable of degrading these compounds, further compromising inhibitor efficiency (Fisk et al., 2015). Consequently, soils with higher SOM contents tend to exhibit a lower inhibitor efficiency. Moreover, a large-scale meta-analysis also revealed a significant negative correlation between SOM content and the mitigation efficiency of chemical nitrification inhibitors (Gao et al., 2021). In this study, although HB and HN soils had similar pH values, both UI and UN were less effective in mitigating N_2_O emissions in the HB soil, which had higher SOM content than the HN soil. This negative relationship between SOM content and inhibitor efficacy was also observed across soils with varying pH levels, suggesting that elevated SOM may exert a suppressive effect on the mitigation efficiency of chemical inhibitors.

Soil texture and WFPS are also important influencing factors of both N_2_O emissions and the inhibitors efficiency (Volpi et al., 2017; Pelster et al., 2021). Previous studies have shown that, within a certain range, higher clay content and elevated WFPS tend to increase N_2_O emissions (Volpi et al., 2017). This is primarily due to the promotion of anaerobic microsites under finer soil particles and high-moisture conditions, which promote incomplete denitrification processes (Balaine et al., 2013; Rochette et al., 2018). Consequently, high clay content and WFPS may reduce the effectiveness of nitrification-targeting inhibitors such as UI and NI, which primarily suppress AOB and nitrification pathways (Sanz-Cobena et al., 2012; Volpi et al., 2017). The findings of our study are consistent with aforesaid perspectives. We observed that increased clay content and WFPS were associated with reduced mitigation inhibitors efficiency, while soil properties that enhance aeration (i.e. high sand content) appeared to improve the mitigation efficiency of inhibitors. In addition, AP and AK have been shown to exert significant influences on soil N_2_O emissions (Mehnaz et al., 2019; Li et al., 2020; Liu et al., 2023b). However, their roles in modulating the mitigation efficiency of inhibitors remain relatively underexplored. These nutrient factors need greater attention in future research to better understand their potential interactions with inhibitors performance under various soil and environmental conditions.

RF analysis revealed that soil physicochemical properties were more important predictors of inhibitors efficiency than microbial community α- and β-diversity (**Figure 6**). Notably, RF models constructed using profiles of nutrient cycling functional gene exhibited higher explanatory weight and model fit compared to those based on soil properties or microbial diversity (**Figure 7**). These results suggest that many abiotic factors may ultimately influence the inhibitors efficiency by modulating microbial functions and altering soil biochemical processes. Previous studies investigating the microbial mechanisms underlying inhibitor performance have primarily focused on key microbial taxa and functional genes (Wu et al., 2018; Lu et al., 2019). In this study, well-established abiotic predictors, such as soil pH, SOM, WFPS, and texture, were identified as significant variables contributing to inhibitors efficiency base on RF model, these factors frequently emphasized in earlier studies (Shi et al., 2017; Li et al., 2020; Liu et al, 2023b), thereby supporting the robustness and consistency of our analytical approach. Furthermore, functional genes related to nitrification (e.g., *amoA*, *nxrA*) and denitrification (e.g., *narG*, *nirS/K*, *norB*), which have been widely recognized for their roles in regulating N_2_O emissions and inhibitors efficiency (Ni et al., 2018; Fan et al., 2019; Luchibia et al., 2020), were also found to be strongly associated with inhibitors efficiency in this study. In particular, the gene (*hao*) of hydroxylamine oxidoreductase, which has often been overlooked in previous studies, exhibited a strong correlation with inhibitors efficiency and was identified as a highly important predictor in the RF model. This underscores its potential as a key functional indicator for evaluating inhibitors efficiency.

In addition, the RF model identified several non-N cycling functional genes as important predictors of NI and UN efficiency, including those involved in carbon fixation/metabolism (*por*, *cooS*, *ppgK*, *acnB* and *glmES*), methane metabolism (*mch* and *mcr*), sulfur cycling (*sat* and *dsrAB*), and phosphorus cycling (*pst*). Based on previous studies on the ecological roles of elements such as C, P, and K, we speculate that these nutrients elements may influence N_2_O mitigation of inhibitors efficiency primarily by limiting growth or death of microorganisms that serve as the direct targets of inhibitors. Nonetheless, current study exploring the influence of non-N nutrient elements on the inhibitor efficiency remains poorly. Further investigation into these interactions is warranted to more comprehensively elucidate the environmental drivers underlying the variability in inhibitors efficiency across heterogeneous soils. Such knowledge will be instrumental in improving the field applicability, functional stability, and agronomic effectiveness of inhibitors under diverse agricultural conditions.

## 5 Conclusions

This study aimed to evaluate the effects of UI and UN on N_2_O mitigation and its relevant regulatory mechanisms across diverse soil types. The controlled incubation experiments eliminate the influence of meteorological and other environmental factors, providing a relatively stable environment to investigate the regulatory effects and mechanisms by which soil abiotic and biotic factors contribute to variations in inhibitors efficiency. Our results demonstrate that mitigation efficacy of UI and UN exhibited evident variation in agricultural soils, and both abiotic and biotic soil factors influence the efficiency of UI and UN. Among the abiotic factors, soil pH, SOM, and texture were identified as critical factors. Microbial community and functional structure diversity showed relatively weak effects on inhibitors efficiency, while partial functional genes involved in N, C, P, and S cycling, particularly those associated with greenhouse gas emissions and microbial growth, emerged as significant factors. This situation is due to these genes can directly affect GHG production or indirectly influence biotic processes through their impact on microbial activity. Therefore, future research should not only explore the interactive effects of other major nutrient element on the efficacy of chemical inhibitors, but also identify strategies to enhance their performance and overall agronomic benefits.

## Data Availability

We have uploaded the Illumina data from this experiment in batches to NCBI. The corresponding BioProject ID follows: PRJNA1322060, PRJNA1324493, PRJNA1327205.
