# Supplementary material for: The effects and mechanism of urease inhibitor and its combination with nitrification inhibitor on nitrous oxide emission across four soil types
Source: Front Plant Sci. 2025 Sep 18;16:1663261. doi: 10.3389/fpls.2025.1663261 (PMC12488586; doi:10.3389/fpls.2025.1663261)
Supplement: Supplementary file 1 [file DataSheet1.docx]

The effects and underlying mechanism of urease inhibitor and its combination with nitrification inhibitor on N_2_O emission in four soil types

Churong Liu^1,2^, Benjie Li^1^, Qihua Wu^1^, Diwen Chen^1^, Wenling Zhou^1^, Junhua Ao^1^

^1^ Guangdong Academy of Sciences, Institute of Nanfan and Seed Industry, Guangzhou 510316, China

^2^ Zhanjiang Research Center, Institute of Nanfan and Seed Industry, Guangdong Academy of Sciences, Zhanjiang 524300, Guangdong, China

**Correspondence**

* Correspondence: junhuaao@163.com

**This file includes:**

**Table S1**

**Fig. S1**

Table S1. Alpha diversity indices (Chao1 and shannon) of genus and KOs levels

| Site | Treatments | Taxonomy (Genus level) | | | |  | Function (KO level) | | | |
| --- | --- | --- | --- | --- | --- | --- | --- | --- | --- | --- |
|  |  | Chao 1 |  | Shannon |  |  | Chao 1 |  | Shannon |  |
| JL | Con | 757.4±33.8 | a | 4.78±0.02 | a |  | 7186.7±15.8 | b | 7.49±0.00 | a |
|  | U | 738.2±14.6 | a | 4.71±0.02 | a |  | 7195.7±53.1 | ab | 7.51±0.00 | a |
|  | UI | 734.2±21.7 | a | 4.76±0.01 | a |  | 7283.7±16.6 | a | 7.50±0.00 | a |
|  | UN | 758.9±14.6 | a | 4.76±0.04 | a |  | 7241.0±20.6 | a | 7.50±0.00 | a |
| HB | Con | 811.8±10.6 | a | 5.05±0.06 | a |  | 7581.3±23.7 | b | 7.56±0.00 | a |
|  | U | 740.6±14.8 | b | 5.07±0.03 | a |  | 7708.7±45.7 | a | 7.57±0.00 | a |
|  | UI | 815.3±32.8 | a | 5.09±0.01 | a |  | 7732.3±33.1 | a | 7.57±0.00 | a |
|  | UN | 783.9±15.9 | ab | 5.10±0.02 | a |  | 7728.7±32.0 | a | 7.57±0.00 | a |
| HN | Con | 786.0±12.8 | a | 5.08±0.04 | ab |  | 7606.7±59.3 | a | 7.52±0.00 | a |
|  | U | 782.5±8.1 | a | 5.01±0.07 | ab |  | 7666.7±22.3 | a | 7.53±0.00 | a |
|  | UI | 784.6±14.1 | a | 5.11±0.03 | a |  | 7622.3±73.1 | a | 7.53±0.00 | a |
|  | UN | 791.8±18.2 | a | 4.97±0.02 | b |  | 7662.0±35.8 | a | 7.53±0.00 | a |
| ZJ | Con | 811.3±15.0 | a | 5.16±0.04 | a |  | 7232.7±26.8 | b | 7.51±0.00 | a |
|  | U | 866.2±11.8 | a | 5.20±0.01 | a |  | 7285.3±43.9 | ab | 7.53±0.00 | a |
|  | UI | 833.2±27.4 | a | 5.12±0.02 | a |  | 7321.7±20.1 | a | 7.53±0.00 | a |
|  | UN | 874.5±20.1 | a | 5.16±0.03 | a |  | 7360.7±14.7 | a | 7.53±0.00 | a |


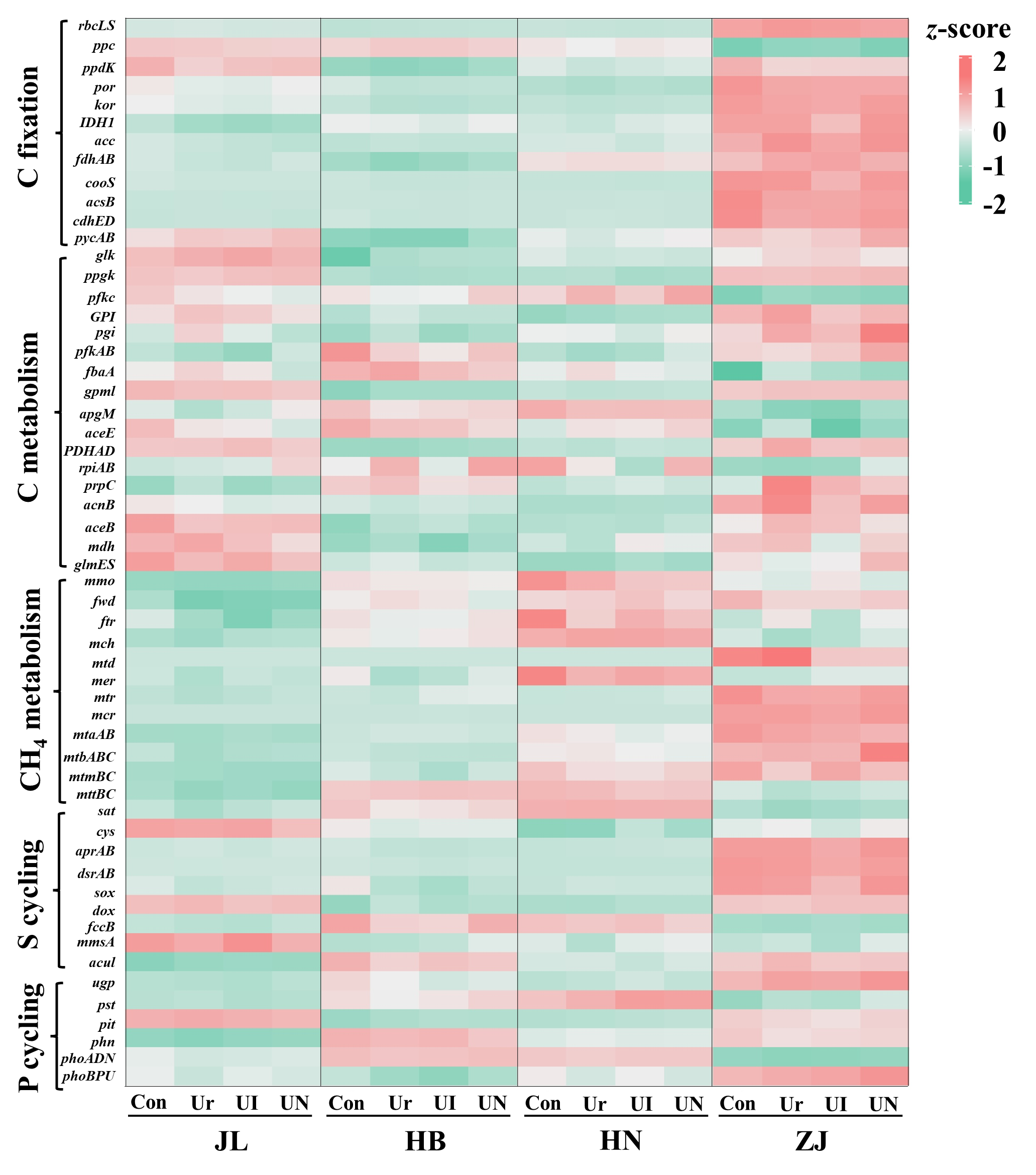


Fig. S1. The change of key functional genes of carbon, methane, sulfur and phosphorus cycling for all treatments.
